# Real-time distribution of pelagic fish: combining hydroacoustics, GIS and spatial modelling at a fine spatial scale

**DOI:** 10.1038/s41598-018-23762-z

**Published:** 2018-03-29

**Authors:** Milan Muška, Michal Tušer, Jaroslava Frouzová, Tomáš Mrkvička, Daniel Ricard, Jaromír Seďa, Federico Morelli, Jan Kubečka

**Affiliations:** 1 Biology Centre of the Czech Academy of Sciences, Institute of Hydrobiology, České Budějovice, Czech Republic; 2 Faculty of Science, University of South Bohemia, České Budějovice, Czech Republic; 3 Faculty of Economics, University of South Bohemia, České Budějovice, Czech Republic; 4 Department of Applied Geoinformatics and Spatial Planning, Faculty of Environmental Sciences, Czech University of Life Sciences Prague, Prague, Czech Republic; 5 Present Address: Fisheries and Oceans Canada, Gulf Fisheries Centre, Moncton, Canada

## Abstract

Understanding spatial distribution of organisms in heterogeneous environment remains one of the chief issues in ecology. Spatial organization of freshwater fish was investigated predominantly on large-scale, neglecting important local conditions and ecological processes. However, small-scale processes are of an essential importance for individual habitat preferences and hence structuring trophic cascades and species coexistence. In this work, we analysed the real-time spatial distribution of pelagic freshwater fish in the Římov Reservoir (Czechia) observed by hydroacoustics in relation to important environmental predictors during 48 hours at 3-h interval. Effect of diurnal cycle was revealed of highest significance in all spatial models with inverse trends between fish distribution and predictors in day and night in general. Our findings highlighted daytime pelagic fish distribution as highly aggregated, with general fish preferences for central, deep and highly illuminated areas, whereas nighttime distribution was more disperse and fish preferred nearshore steep sloped areas with higher depth. This turnover suggests prominent movements of significant part of fish assemblage between pelagic and nearshore areas on a diel basis. In conclusion, hydroacoustics, GIS and spatial modelling proved as valuable tool for predicting local fish distribution and elucidate its drivers, which has far reaching implications for understanding freshwater ecosystem functioning.

## Introduction

The spatial heterogeneity of resources and environmental conditions is an essential property of all biological systems at different spatiotemporal scales^1^. This phenomenon is fundamental to population dynamics, community stability and reflects in animal spatial distribution through the optimized strategies in habitat use for increasing fitness of individuals^2^. In aquatic ecosystems, fish spatial organisation and investigations of its drivers and dynamics gained much interest after global fisheries crises^3^ and in context of predicting impact of global change^4^. The spatial distribution of freshwater fish is shaped by extrinsic and intrinsic cues, i.e. physicochemical parameters^5^, resource availability^6^, predator-prey interaction^7^, intraspecific relationships^8^ and individuality in habitat use^9^. Knowledge of spatial distribution patterns of fish, an important component in freshwater ecosystems, and their direct and indirect effect need to be taken into account for a thorough understanding of ecosystem functioning. Moreover, when fish species are of commercial or conservation interest understanding and predicting of these patterns becomes essential requirement for sound management^10^.

Spatial distribution of pelagic fish has long been assumed to be relatively homogenous both in time and space in freshwater ecosystem in comparison with organisation of components in other ecosystems or littoral areas^11,12^. In lakes, more recent studies examined the heterogeneous nature of fish distribution in this habitat^13^. However, most of the studies have been attempted at lake or reservoir scales showing the response of fish to large-scale patterns, e.g. nutrients, temperature and turbidity gradients^14,15^. On the contrary, investigations made at a fine-scale, with high resolution and taking into account the local environmental conditions are really scarce. The reason can be that changes of spatial distribution of highly mobile organisms are assumed to be more frequent and much faster on the local-scale than at the large-scale because are responding to the faster and local specific changes of resource availability^16^. Determination of the driving environmental factors of pelagic fish spatial distribution at this local-scale has not been sufficiently clarified so far, however the local processes are of great importance for fish behaviour, predator-prey interactions and food web structure and stability^17,18^.

Fish as predators in freshwater ecosystems play a crucial role in establishing ecosystem stable states through the cascading trophic interactions^19^. Specially, pelagic fish as highly mobile consumers of zooplankton stabilize the lake food web by ranging over larger area than their zooplankton prey. Hence, changes in pelagic fish distribution can be a major driving force in phyto-zooplankton dynamics and emphasize the importance of knowledge of their occurrence patterns. Additionally, these mobile consumers often cross habitat boundaries and link food webs between habitats^20^. A fine-scale model of fish occurrence made in appropriate temporal detail could help to determine and assess the extent of fish foraging interactions and habitat coupling, thereby estimating their influence in establishment of ecosystem stable state.

Ideal tool for observations of spatial distribution of organisms in aquatic habitats represents hydroacoustic methods^21^. It is well-established and recognized for quantitative and real-time observation of undisturbed fish populations with high spatiotemporal resolution^22^. Together with the expansion of Geographic Information Systems (GIS) it creates an ideal opportunity for mapping and modelling the spatial distribution of pelagic fish^23^. However, only a few attempts to model the real-time relationship between pelagic fish occurrence and relevant environmental variables have been made until now in freshwater ecosystems^24,25^.

The ability to quantify spatial structure patterns depends on the degree of understanding its spatial autocorrelation. This phenomenon is present in most ecological datasets and plays a crucial role in the analysis of the spatially-explicit data^26^ because it violates the assumption of independent samples of most standard statistical procedures^27^ that may lead to spurious significance tests and exaggerate resource preferences^28^. The appropriate model based approach can describe the spatial autocorrelation of the phenomenon of interest, and furthermore certain parameters of the spatial model can reveal spatial patterns of organism distribution^29^.

This study aimed to assess day- and nighttime horizontal spatial distribution of pelagic fish at local (fine) scale and to identify determinants for such patterns. The study focused on a part of Římov Reservoir, Czech Republic, which was extensively sampled at a detailed spatiotemporal scale (>42 % area, every 3-h) during two days. Then, the distribution of fish biomass was modeled in relation to environmental variables. Specific objectives included determining whether the fish preferences could be generalized during day- and nighttime periods or between different days and nights.

## Results

### Fish spatial structure

**Figure 1:**
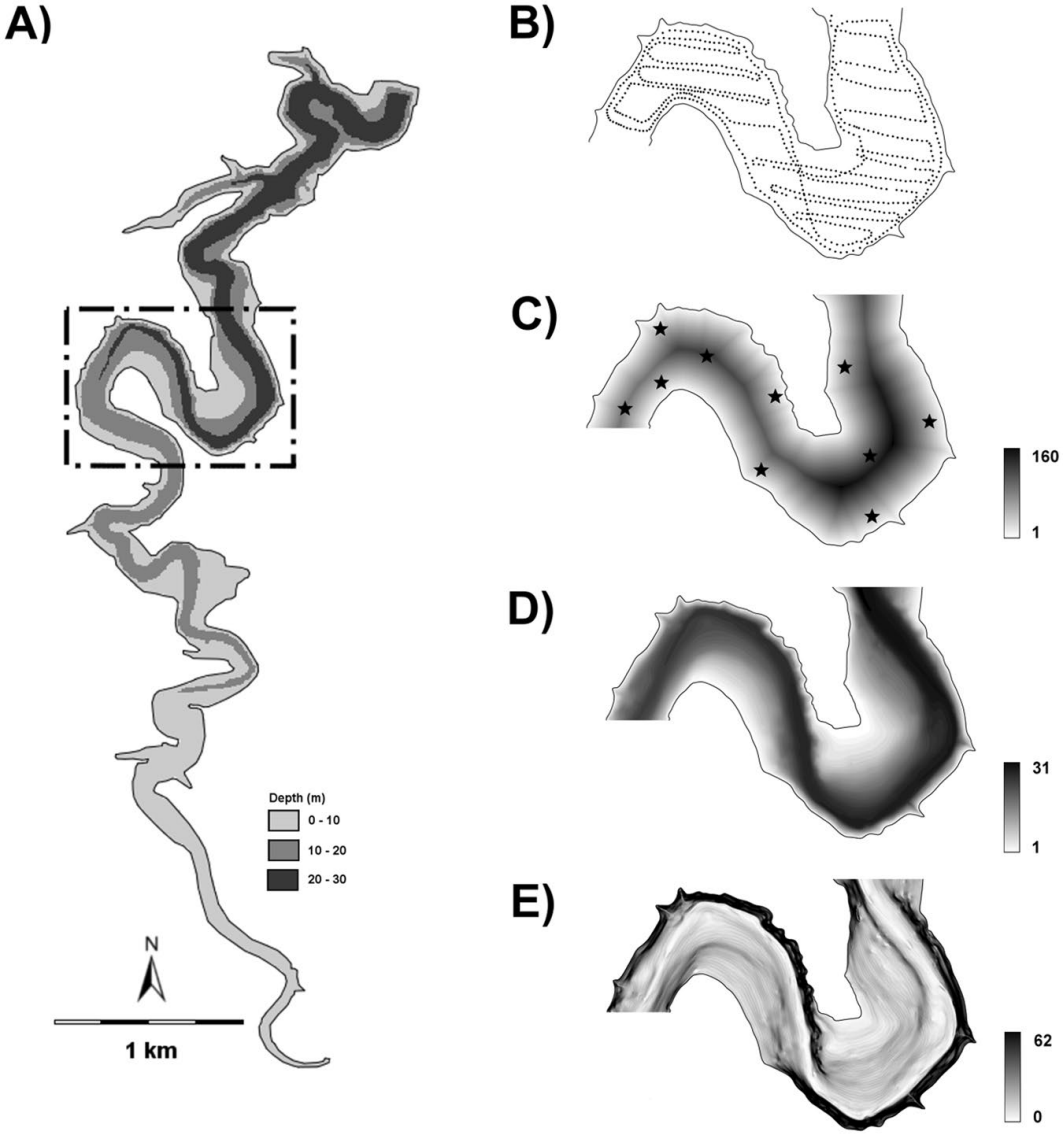
Bathymetric map of the Římov Reservoir showing highlighted study area (**A**). A representation of sampled points of hydroacoustic surveys (**B**) and visualization of used environmental covariates, distance from the bank (m), depth (m), bottom slope (degrees) (**C**–**E**). Zooplankton sampling sites are depicted by stars in (**C**). The figure was generated by the software ArcMap, version 10.3. (http://www.esri.com/).

**Figure 2:**
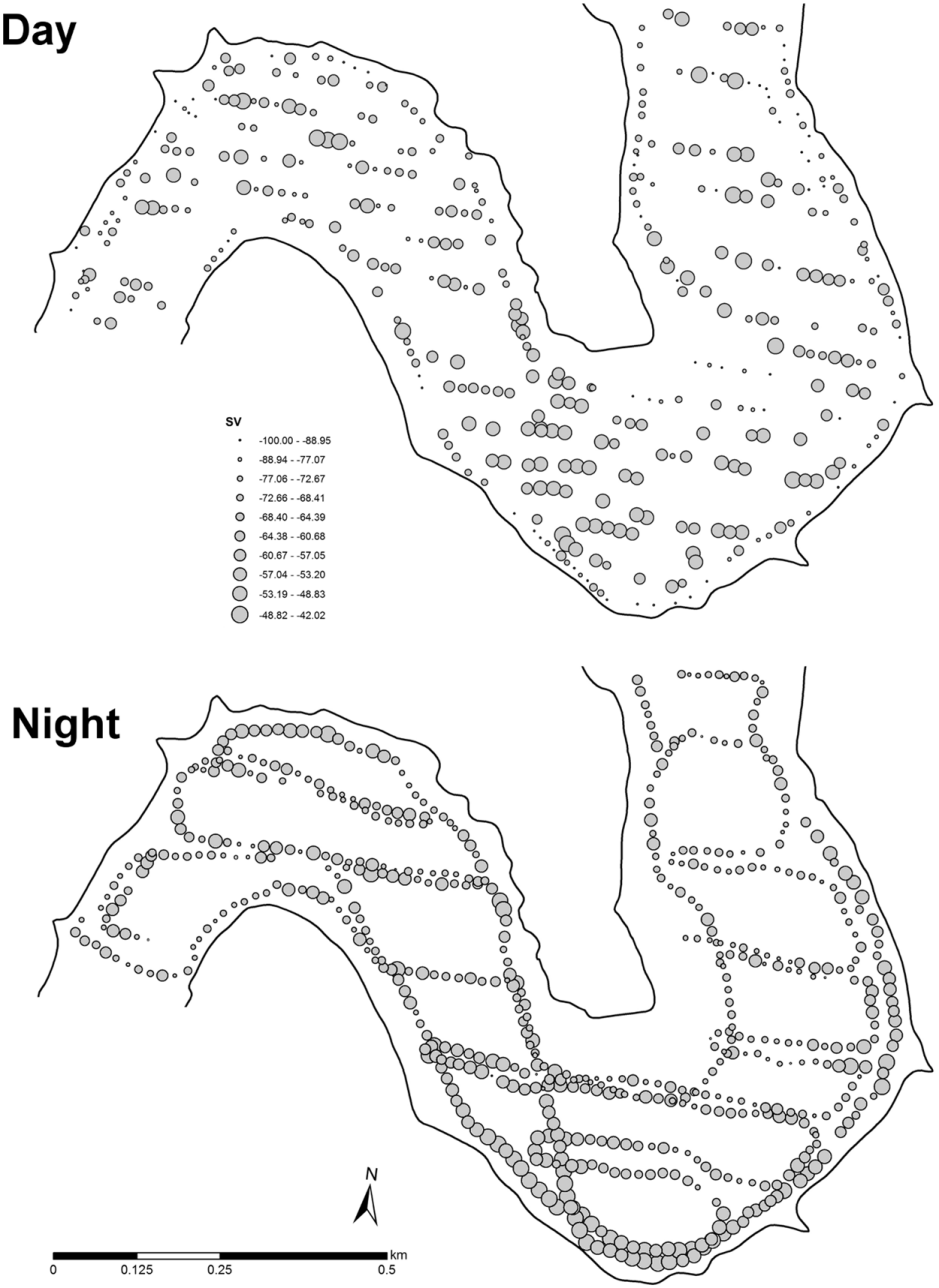
Map of spatial variation in fish biomass at day- and nighttime. Only survey number 15 (day) and 10 (night) were chosen for example, the pattern in other particular surveys was similar. Fish biomass is expressed as volume backscattering strength (S_V_, −dB). The figure was generated by the software ArcMap, version 10.3. (http://www.esri.com/).

**Figure 3:**
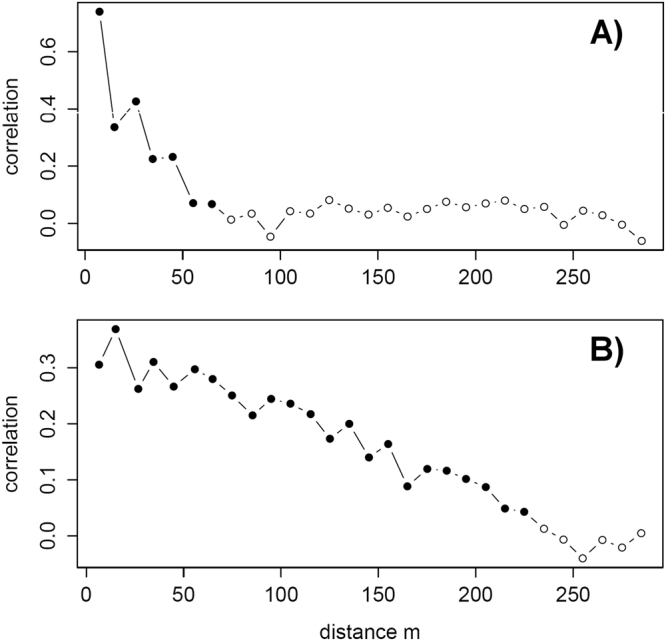
The correlogram (plot of autocorrelation versus distance lags) of selected day (**A**) and night time (**B**) surveys. The filled points specify the significant correlations, where the significance is taken on the level 0.05 and it is computed via 1000 bootstrap permutations.

In pelagic area of the Římov Reservoir, the overall 13 hydroacoustic surveys produced a total of 8226 transects with estimated fish biomass coupled with environmental covariates (Fig. 1). The average pelagic fish biomass was similar during day and night, 141 and 131.5 kg ha^−1^ respectively. The local-scale spatial structure of pelagic assemblage was not homogeneous (Fig. 2) and fish biomass was positively autocorrelated in all particular surveys during both days (Moran’s *I* 0.26–0.51; p < 0.001; Table 1). Similarly, correlograms of fish biomass exhibited significant spatial structuring decreasing with distance between sampling points, meaning that fish were markedly aggregated instead of randomly dispersed (Fig. 3). The shape of the correlogram curves differed between day and night suggesting different degree of aggregation in fish spatial structure. Fish were more aggregated during day, showing high serial autocorrelation at close distances (Fig. 3A), while fish displayed more dispersed spatial structure at night (Fig. 3B) however spatial autocorrelation was always statistically significant (Table 1).

**Table 1:** Calculated Moran’s *I* spatial correlation coefficient for 13 surveys in the raw data and in spatial-lag regression model residuals.

	**data**		**residuals**	
	**Moran’s I**	P-value*	**Moran’s I**	**P-value**
G1	0.465	p < 0.001	−0.37	p = 0.14
G2	0.379	p < 0.001	−0.036	p = 0.16
G3	0.308	p < 0.001	−0.0254	p = 0.246
G5	0.26	p < 0.001	0.026	p = 0.21
G6	0.377	p < 0.001	0.005	p = 0.39
G7	0.29	p < 0.001	0.046	p = 0.055
G9	0.401	p < 0.001	−0.048	p = 0.87
G10	0.475	p < 0.001	0.066	p = 0.026
G12	0.51	p < 0.001	0.029	p = 0.16
G13	0.45	p < 0.001	−0.0123	p = 0.354
G14	0.465	p < 0.001	−0.0141	p = 0.323
G15	0.427	p < 0.001	−0.0238	p = 0.17
G16	0.313	p < 0.001	0.037	p = 0.12

^*^P-values were calculated by the permutation test.

The abiotic factors showed low variability in close distances (0–20 m) and reached maximum variability between 40–50 m (see Supplementary Fig S1). The analysis of environmental parameters and fish occurrence highlighted the general environmental preferences of pelagic fish as well. All observed environmental parameters were statistically significant in the spatial-lag regression model (Šidák’s multiple comparison test, Day P < 0.0064; Night P < 0.01 at α = 0.05; Table 2). On the contrary, the Moran’s scores were not significant in any residuals from all particular spatial-lag regression models, showing that autocorrelation was treated correctly and that the model fitted the data adequately^30^.

**Table 2:** Spatial Lag models calculated with fish biomass as response variable and distance to the bank (DTB), underwater light intensity, bottom slope, depth as independent variables.

Survey no.	DTB		Light		Depth		Bottom slope		adjusted R^2^
	Coefficient	p - value	Coeficient	p - value	Coeficient	p - value	Coeficient	p - value	
1	−0.042	0.003*			0.360	0.000*	0.147	0.000*	0.24
2	−0.030	0.007*			0.122	0.005*	0.082	0.011	0.24
3	−0.060	0.000*			0.001	0.986	0.011	0.684	0.18
5	0.017	0.329	0.001	0.714	0.014	0.038	−0.175	0.002*	0.12
6	0.020	0.466	0.069	0.000*	0.263	0.006*	−0.191	0.005*	0.19
7	0.044	0.016	0.003	0.077	0.184	0.003*	0.032	0.509	0.15
9	−0.027	0.101			0.079	0.223	0.098	0.002*	0.24
10	−0.037	0.003*			0.065	0.163	0.067	0.043	0.27
12	0.027	0.115	0.005	0.650	0.116	0.072	−0.025	0.574	0.35
13	0.076	0.000*			0.129	0.042	−0.002	0.972	0.34
14	0.079	0.000*	0.001	0.317	0.076	0.207	0.081	0.120	0.36
15	0.048	0.000*	0.004	0.000*	0.068	0.251	−0.053	0.206	0.31
16	0.066	0.000*	0.005	0.001*	0.053	0.153	−0.051	0.039	0.27
Day-time rate of significance		0.0007		0.0093		0.0415		0.0651	
nighttime rate of significance		0.0011				0.0071		0.0145	

Significant factors, according the adjusted p-value after Šidák’s correction (to the critical value of significance α = 0.05 is 0.0064 for day-time and 0.01 for nighttime models) are labelled with asterisk.

### Daytime fish preferences

**Figure 4:**
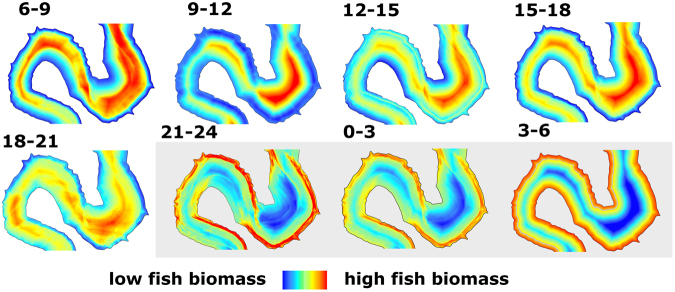
Visualization of Spatial Lag model showing development of spatial distribution patterns of pelagic fish biomass during 24 h. Fish biomass is expressed as volume backscattering strength (S_V_, −dB). The scale differs between particular periods and therefore is not specified. Night patterns are presented on grey background. The figure was generated by the software ArcMap, version 10.3. (http://www.esri.com/).

The pelagic fish spatial structure was spatially consistent during both days and across all the 8 daytime surveys. The fish preferences to particular environmental parameters was temporarily stable in all daytime surveys during both days and differed constantly in most parameters from opposite nighttime patterns (Table 2). The diel period was the most important factor influencing spatial structure of fish. The main driver of fish spatial distribution was distance from the bank (DFB; Table 2) showing that fish preferred more distant areas from the shore during the day. The fish occurrence was positively related to intensity of underwater light and depth. On the contrary, bottom steepness was negatively related to fish biomass with the smallest rate of significance. Models developed for daytime fish distribution explained a significant part of the fish occurrence variability (Table 2; mean R^2^ = 0.24; max. 0.37). The realisation of fish spatial distribution models with mixed effect of all covariates through the whole daytime is depicted in Fig. 4. The general model of fish spatial structure pattern during daytime indicates that most fish were aggregated in the central, deep and highly illuminated areas of the reservoir.

### Nighttime fish preferences

Nighttime distribution of fish was spatially consistent in all surveys during both nights. Similarly to daytime, the DFB was still the most important environmental driver influencing the fish spatial distribution at night, while it was related negatively to the fish occurrence, indicating high fish affinity to the nearshore areas during the night (Table 2). Depth and bottom slope were positively correlated with fish occurrence, the former with higher importance than the latter. For the night spatial-lag models, coefficients of determination were similar to those from the daytime (Table 2; mean R^2^ = 0.23; max. 0.27). The realisation of nighttime fish biomass models is shown in Fig. 4. The overall trends in nighttime fish preferences show that fish were more concentrated near the steep sloped banks above higher depth.

**Figure 5:**
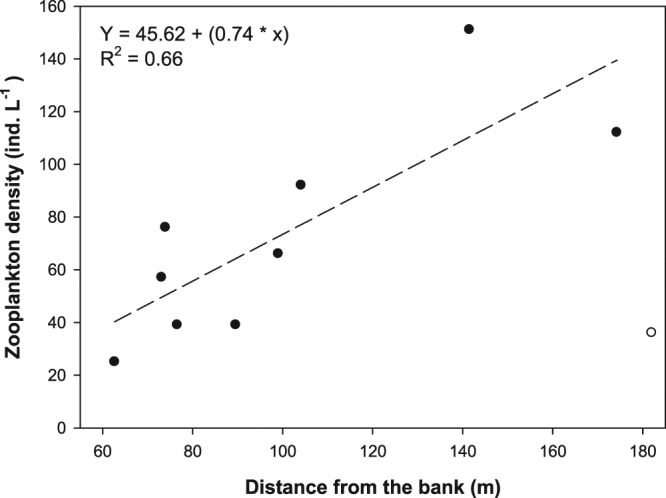
Epipelagic zooplankton density (ind. L^−1^) plotted against distance to the shore (m). The one outlier value is depicted with unfilled dot.

The model for the survey from 0600–0900 did not show any significant relationship with any covariates. This time period overlaps with the transitional period between creation of stable nocturnal and diurnal fish distribution patterns. Epilimnetic zooplankton was distributed quite heterogeneously, attained densities ranging from 63 to 182 ind. L^−1^ (CV = 0.38; mean 107) at the ten sampling sites and was dominated by large cladocerans. A significant relationship between zooplankton density and DFB was found (F (1; 8) = 13. 34, p = 0.008, R^2^ = 0.66) if one outlier sample with low zooplankton density in the high distance from the bank was excluded (Fig. 5).

## Discussion

In this study we present the first attempt to develop a model of pelagic fish spatial distribution at a local-scale with high resolution and spatiotemporal coverage that explains and predicts the distribution patterns during whole diel cycle and in relation to environmental factors in freshwater ecosystem. The spatial-lag regression model allowed us to evaluate dependence of fish occurrence on local abiotic predictors without effect of spatial autocorrelation and we used these relationships to predict fish occurrence at any unobserved location of the study area.

Our findings revealed that the daytime spatial structure of pelagic fish, which has been supposed as homogenous based on the direct catching methods^31^, is on the contrary highly heterogeneous with high degree of spatial autocorrelation. High spatial autocorrelation and its sharp decrease with distance in correlograms correspond to the common occurrence of relatively small aggregations of fish (shoals <20 m in diameter). Our results are therefore consistent with few studies that documented fish aggregations of comparable size (i.e. 10–17 m) in pelagic areas of lakes and reservoirs with similar^32,33^ or coregonids dominated fish assemblage^34^ and moreover provide more detailed and concrete characterisation of pelagic fish organisation. Creation of shoals has been shown as frequently occurring general pattern of fish behaviour ensuring an effective strategy to reduce predation risk^35^ and increase exploitation of patchy food resources in unstructured pelagic habitat^36^. The nighttime fish distribution was much more homogenous in comparison with daytime, but still not fully random. This night homogenization is expected because fish use vision as the primary sense to maintain shoals^37^ and light levels at night are simply too low for them to visually maintain contact with shoaling neighbours. On the other hand, fish still preferred specific areas close to the steep banks at night; however, their aggregations were more diffused.

Surprisingly, the daytime fish distribution was significantly spatially structured even at further distances (up to 70 m), which suggests to another level of spatial structuring. This pattern is most likely related to the organisation of clusters of shoals and it indicates spatial structuring of fish in reservoirs at least at three levels. The first corresponds to the above discussed fine-scale structuring of individual fish into shoals^34^, the second represents organisation of shoal clusters in preferred pelagic areas at scale of tens of meters and the last corresponds to the organisation along coarse-scale gradients at reservoir level^15^. The presence of spatial structures at several levels may occur because of combination of biotic interactions and common response to environmental forcing acting at different scales^38^.

The dynamics of pelagic fish distribution patterns is expected to be very fast on small scale, because freshwater pelagic fish are very mobile with sustained swimming speed up to 40 cm s^−1 ^^39^. Despite these consideration, our results revealed noticeable stability of pelagic fish spatial organisation during both days and both nights with consistent fish preferences for particular covariates. Similar persistence in spatial patterns of fish distribution has been further shown in terms of day-to-day variability^40^, pelagic assemblage species composition^41^, overall biomass and density^42^ and between years variability^31^ in temperate lakes and reservoirs during summer. The evidence above and our results suggest that while our experiment was limited in observed time period of two days, we were able to capture the bulk of spatial distribution dynamics of pelagic fish assemblage in reservoir and the observed pattern represents the general summer situation. The only nonsignificant results of fish preferences were observed during survey carried out between 0600 and 0900. This time period overlaps with highly unstable spatial structure characterized by the morning peak of fish activity^43^ when major movements between nocturnal and diurnal habitats occurs^44^.

The pelagic fish showed clear and temporally stable daytime preferences for central, deep and highly illuminated local areas of the pelagic habitat within the reservoir. This particular finding provides new and more detailed information to the described general daytime preferences of adults of the most common fish species in Europe (i.e bream, roach, and perch) to the pelagic habitat in general which has been observed in several studies from lakes and reservoirs^31,45,46^. Since the dominant pelagic species in temperate reservoirs have been shown as almost exclusively zooplanktivourous^47^, and our results showed increasing zooplankton density towards central areas within the pelagic habitat, fish most probably followed the food gradient and utilized the higher food-profitable central places of pelagic habitat in Římov Reservoir similarly as in other lakes^48^. On the contrary, the direct effect of locally abundant fish depleting by its feeding the zooplankton densities was observed at a local scale^18^. Unfortunately, our zooplankton sampling strategy did not allow observing the local scale distribution changes and therefore direct evaluation of fish and zooplankton spatial relationship on the local scale was not possible. However, the appropriate zooplankton sampling undertaken simultaneously and with the same spatiotemporal resolution as fish sampling would allow incorporating this important biotic factor and significantly improve the explanatory power of fish spatial models in future.

Fish preferences and exploitation of pelagic habitats as the structurally simple environment with little protection has been shown related to the threat of piscivore predation as well^49^. Numerous studies documented serious effect of predation threat to the distribution of vulnerable, mostly small-sized, fish in freshwater lakes and reservoirs when these fish generally avoided pelagic habitat and occurred exclusively in safer inshore habitats^7,31^. On the contrary, our observations of pelagic fish preference, mostly represented by large adults, for potentially dangerous central parts of pelagic areas suggests its lower predation vulnerability from occurring gape-limited predators^50^ which is probably further enhanced by its shoaling behaviour^51^.

Furthermore, we revealed significant importance of underwater light on local fish distribution within the pelagic habitat. This new finding showing the advantage of fish occurrence at highly illuminated places corroborates previous laboratory experiments demonstrating a consequence of increased reaction distance and better detectability of large zooplankton individuals or zooplankton patches under high contrast conditions^52^. The importance of light intensity for foraging of dominant pelagic cyprinids and perch, which are visually orientated particulate feeding or gulping zooplanktivores, has been implied by its exclusive daytime feeding activity^47^ and performance of sinusoidal swimming, a special light-induced searching behaviour pattern, in temperate reservoir under natural condition^39^. Further, the essential importance of high light intensity for detection of zooplankton has been demonstrated by a significant decrease in the proportion of individuals performing sinusoidal swimming as a consequence of even small decrease of solar radiation, caused by crossing clouds^53^. On the other hand, the contrasting effect of fish aggregation within places of lower light intensities (i.e. highly turbid water) at whole lake scale in Upper Lake Constance has been shown by Heege and Appenzeller^54^ and similarly Jacobsen *et al*.^55^ showed increased occurrence of fish in pelagic habitat in turbid lake in contrast to clear lake. Their findings were confirmed in controlled laboratory experiments showing turbid water as a hiding place, where zooplanktivourous fish decrease their visual contrast (i.e. detectability) but still can effectively feed on zooplankton^56^. In this view, our results suggest that maintaining higher feeding efficiency in highly illuminated localities is more important factor than potential predation risk for adult pelagic fish distribution.

The daytime fish aggregation above gentle sloped bottom and higher depths was unexpected result and can probably refer to the general fish preference of mild sloped habitats^57^. The fish preference for deeper areas is probably a consequence of necessity of sufficient manoeuvring space for sinusoidal swimming, during which fish swim up and down with maximum amplitude greater than 3 m^39^. Moreover, the majority of fish biomass was observed during the summer season aggregated in the surface epilimnetic layer and only a negligible amount of fish occurred in deeper habitats^58^ which makes the mechanism underlying preference for surface layer above deeper areas not so evident.

Our results highlighted the diurnal cycle as highly important factor for spatial distribution of pelagic fish that supports previous studies describing diurnal migrations between littoral and pelagic areas using direct catches^31^. Moreover, our findings clearly showed differences in local-scale fish spatial organisation between day and night and particular preferences for close areas to the steep shore and above higher depth at night. This apparent switch between day and night fish preferences is probably a consequence of the common horizontal movements of pelagic assemblage close to the shore at night^6^. Part of the daytime pelagic community migrates to the shallow inshore areas^31^, that has been demonstrated as important nighttime resting habitat especially for perch^59^, whereas other species still create substantial biomass in pelagic areas^60^ and move only closer to the steep sloped shore. This movement close to the structured littoral is probably inherited in the part of the originally riverine fish population^11^ and steep sloped banks are characterised by relatively high structural complexity with only a few meter distance to the prefered daytime pelagic habitat. Thereby, fish can spend the nighttime relatively close to the bank with minimal travel expenditures connected with migration to the daytime feeding habitat in pelagic areas.

Most of studies conducted on the relationship between pelagic fish distribution and environmental parameters used hydroacoustics^18,24,54,61^ as a fast, robust, quantitative and non-selective method^62^. These merits are unfortunately put at disadvantage in terms of impossibility of species identification in multi-species assemblage. Modelling multi-species distribution data generalize habitat preferences of several species with possibly gently dissimilar ecological demands that implies additional variability in the model. Hence, multi-species models have been shown that they have smaller explanatory power in comparison to similar single-species model^63^. Inspite of this, multi-species studies represent in freshwater lakes and reservoirs the only opportunity to study pelagic fish spatial structure and still explains significant amount of variability, similarly like in our model^24,61^.

Finally, it is essential to state that correlation of spatial distribution patterns with environmental covariates is dependent on the selection of appropriate variables, its nature and sampling density. Our results showed that observing fish spatial structure on a fine-scale can help us to describe and explain local heterogeneity previously summarised during large-scale sampling methods^31^. Further, we demonstrated that detailed hydroacoustic surveys in combination with easily available local environmental parameters (DFB, depth, light intensity, bottom slope) and appropriate spatial modelling approach can explain a significant part of variability in pelagic fish distribution patterns in reservoirs and is probably easily transferable. Certainly, increasing the number of relevant covariates, especially more dynamic biotic parameters (e.g. zooplankton or predator density) covering the similarly detailed spatiotemporal scale as hydroacoustic data can significantly improve the prediction performance of spatial models in the future. Incorporation of internal triggers connected with population size, age structure, fish conditions, species diversity and behaviour that are too complex to be included in current distribution models can be helpful in the future. We consider these results of primary interest not only from the ecological point of view but also to the aims of fisheries management, as the understanding of the effects of environmental factors on fish distribution patterns may represent key information able to improve significantly the quality of scientific advice on exploited fish populations.

## Materials and Methods

### Study area

The study was undertaken in the meso−eutrophic Římov Reservoir (48°51′N, 14°29′E; South Bohemia, Czech Republic) during 7–9 August 2007. The reservoir represents a canyon−shaped water body with narrow (max. width 600 m), elongated shape (length 10 km) and alternating slight and strong sloped banks (Fig. 1). The reservoir surface area was 162 ha during the study. The reservoir is dimictic, with summer stratification apparent from April to October. The average retention time of this water body is approximately 75 days. The pelagic fish assemblage in the Římov Reservoir was investigated by purse seining during experiment and was represented by bream, *Abramis brama* (L.), roach, *Rutilus rutilus* (L.), bleak, *Alburnus alburnus* (L.)) and perch, *Perca fluviatilis* L.^60^. Predatory fish (asp, *Leuciscus aspius* (L.), pikeperch, *Sander lucioperca* (L.), pike, *Esox lucius* L. and European catfish, *Silurus glanis* L.) represent an important proportion (14.5 % of biomass) of the assemblage. The study site, situated in the middle part of the reservoir, represented 26 % (42 ha) of total reservoir surface (Fig. 1A).

### Abiotic factors

Topographic and *in situ* measurements were used in this study to clarify the relationship among environmental predictors and fish distribution. All values of the DFB, depth and slope were derived from the raster layers based on intersection of hydroacoustic transect’s centre of gravity and corresponding layer in GIS. All the layers were prepared in ArcMAP 10, Spatial Analyst extension (ESRI Inc., CA, USA) with 1 m cell size. The DFB raster layer was created by interconnection of two multiple buffers created at 1 m distance from left and right banks and the maximum DFB in the study area was 320 m (Fig. 1C). The depth and slope raster layers were made from digital elevation model (DEM) of the Římov Reservoir calibrated to the water level during experiment. The depth raster was made by reclassification of DEM when actual water level was set as 0 and increased with 1 m step to lower elevations. The slope raster was calculated by slope tool (in ArcMAP 10, Spatial Analyst) in degrees. Maximum and mean depth was 31 and 16 m respectively (Fig. 1D) and the bottom slope attained values up to 62° (Fig. 1E). The underwater light intensity (µE m^−2^ s^−1^) was measured 1 m under the water level with the LI-1400 dataloger with LI-193 sensor (LI-COR Biosciences, Nebraska, USA) at 1 s interval.

### Hydroacoustic sampling

The acoustic study was made by a combination of a horizontally orientated elliptical transducer (ES120_4; nominal beam angles 9.2° × 4.3°) and a circular transducer (ES120_7C; nominal angle 6.4°) aimed vertically. Both transducers were operated by a SIMRAD EK 60 split-beam echosounder at a frequency of 120 kHz via a multiplexer. The elliptical transducer was orientated starboard and tilted 4° downwards. The echosounder was driven by the SIMRAD ER 60 software (version 2.2.0), a pulse duration of 128 μs was constant and the ping rate was set at 5 pings s^−1^. Before the survey, the whole system was calibrated using a 23 mm diameter copper calibration sphere (target strength (TS) −40.8 dB) according to^64^.

The acoustic survey was performed along a predesigned dense parallel grid (Fig. 1B) with a constant speed of 1.5 m s^−1^ and within 3-hours interval during two consequent 24-h cycles. A total of 16 hydroacoustic surveys was performed in total. Three surveys (0600–0900 and 1800–2100 during the first day and 0300–0600 on the second day) were unfeasible to processed. Each survey measured 11.5 km and trajectory was mainly concentrated to the pelagic areas with variable depths and bottom slopes. The survey was restricted to depths >2 m. At this depth the recording was filled with bottom echoes and acoustic data were unreliable. The position of the survey boat was measured using a Garmin GPSMAP 60CSx GPS receiver with an external antenna attached to the transducer’s holder at the fore part of vessel and the geographic coordinates were embedded into the acoustic data files.

Raw acoustic data were converted and analyzed with the Sonar5 Pro post-processing software (version 5.9.1, Lindem Data Acquisition, Oslo, Norway). Because the overwhelming majority of fish was present in the surface layer (between surface and 4 m) and only a negligible amount of fish occurred at greater depths i.e. in vertically recorded data^60^, the vertical data were not further considered.

The horizontal recordings were bound by setting the upper and lower limit of the pelagic layer at 4 and 20 m from the transducer, respectively. These limits were set to avoid a bias caused by the transducer near-field (2.29 m) and far-field non-spherical spreading at the thermocline or surface layers. A manually-defined bottom line was used in order to exclude noisy parts in a record or bank echoes occurring within the pelagic layer and only data within the pelagic layer were processed. In addition, Cross Filter Detector^65^ was used to eliminate noise in the horizontal data with the following parameters: foreground filter: height 5 and width 1, background filter: height 55 and width 1, offset +6 dB, perimeter length: 10–10 000 (Nr. of samples around the detected region), ratio: min 1 - max 270 (track length/mean echo length), max intensity: (−60 to −10 dB).

Non-fish echoes were eliminated by setting a −65 dB minimum TS threshold. All targets exceeding this threshold were echo-integrated within 15 m long transects in order to reveal fine spatial distribution changes. Obtained volume backscattering strength values (S_V_; −dB) were georeferenced with the transect’s centre of gravity, merged with the corresponding values of environmental covariates and projected to metric S-JTSK coordinate system.

Fish were sampled by hydroacoustic methods only and these methods represent a non-invasive, remote sensing, approach. Any life fish were not handled during the experiment and sampling was conducted in accordance with EU guidelines^66^.

### Zooplankton sampling

Samples of epipelagic zooplankton were collected on 8^th^ August 2007 at ten localities located randomly within the study site (Fig. 1C). At each locality, two vertical hauls through the epilimnion were taken (net diameter 20 cm, mesh-size 200 µm) from depth of thermocline (5 m) during the daytime. Sampling was reduced to epilimnion and daytime because dominant zooplankton (*Daphnia* sp.) occurs there principaly and no diel changes were observed in Římov Reservoir^67^. Catches were pooled into one sample and immediately preserved in 4% formaldehyde. In the laboratory, zooplankton densities were estimated by the standard method of microscopic counting in a Sedgewick-Rafter chamber.

### Statistical analysis

Environmental covariates were examined for univariate Pearson Product-Moment correlation and probability of correlation was <0.2 in all cases. Regression (zooplankton density and DFB) and correlation analysis were carried out in the STATISTICA software package ver. 9.1. (StatSoft, Inc., 2010). The variograms of environmental covariates has been computed in R package “fields”^68^. The spatial regression analysis was performed in the OpenGeoDa 1.0.1 software^30^. According to spatial diagnostics (Lagrange multiplier test statistics), we chose the spatial-lag regression model to analyse the data^30^. The full model was:

1 $$SV=\rho W\,Sv+{\beta }_{1}\,Light+{\beta }_{2}\,DBF+{\beta }_{3}\,Bottom\,slope+{\beta }_{4}\,Depth+\varepsilon $$

here W express a weight matrix of spatial distances and ρ measures the degree of the spatial correlation. Together the term ρ W Sv measures spatial spill–over effect which occurs in Sv. ε is an error term and β_i,_ i = 1,…, 4 are regression parameters.

The spatial autocorrelation of the data and spatial-lag regression model residuals were evaluated by Moran’s *I* statistic^69^. The index quantifies the degree of spatial autocorrelation between observations. We used the permutation test to examine whether the Moran’s *I* statistic is equal to 0. The spatial regression analysis is a refined regression method which takes into account the spatial correlations of the data. The spatial correlations are summarized in a spatial weight matrix, computed on the basis of distance. For determination of appropriate distance, we examined the range of spatial autocorrelation in data for various distances by correlogram analyses made in R 3.2.2.^70^ using package spdep at the 0.05 significance level and computed via 1000 bootstrap permutation^71^. According to the shape of the correlogram curves, several spatial regression model trials, with different lag distances (50 m, 30 m, 20 m) and subsequent checks of spatial autocorrelation in the model residuals were performed. Since the residuals did not show any spatial autocorrelation (no significant permutation test for the Moran’s *I* statistic) and autocorrelation decreased with distance in correlograms, we assume a distance of 20 m to be appropriate and the spatial-lag model to be correctly chosen.

### Summary statistics

Since we have several independent realizations of the data and thus several p-values are produced from the spatial-lag regression models for one covariate, we used the Šidák correction to adjust the critical value of significance^72^. For evaluation of the importance of particular covariate influence, we used the logarithmic mean of the p-values to summarize the information about the rate of significance. The logarithmic mean p-value was calculated from the sample of p-values p_i_, i = 1, .., n by:

2 $$Lp=Exp\,((Ln(p)+\ldots +Ln(pn))/n)$$

## Electronic supplementary material

Supplementary information
