# Supplementary material for: Real-time distribution of pelagic fish: combining hydroacoustics, GIS and spatial modelling at a fine spatial scale
Source: Sci Rep. 2018 Mar 29;8:5381. doi: 10.1038/s41598-018-23762-z (PMC5876353; doi:10.1038/s41598-018-23762-z)
Supplement: Supplementary file 1 — Supplementary information [file 41598_2018_23762_MOESM1_ESM.docx]

**Supplementary materials for manuscript:**

Muška, M., Tušer, M., Frouzová, J., Mrkvička, T., Richard, D., Seďa, J., Morelli, F., Kubečka, J.: Real-time distribution of pelagic fish: combining hydroacoustics, GIS and spatial modelling at a fine spatial scale

**Supplementary Figure S1.** Variograms describing variability of abiotic factors (A - depth, B - distance from the bank, C - bottom steepness) dependent on distance.

**B**

**C**

**A**

**A**
